# Plasma neurofilament light chain concentration in the inherited peripheral neuropathies

**DOI:** 10.1212/WNL.0000000000004932

**Published:** 2018-02-06

**Authors:** Åsa Sandelius, Henrik Zetterberg, Kaj Blennow, Rocco Adiutori, Andrea Malaspina, Matilde Laura, Mary M. Reilly, Alexander M. Rossor

**Affiliations:** From the Department of Psychiatry and Neurochemistry (Å.S., H.Z., K.B.), Institute of Neuroscience and Physiology, Sahlgrenska Academy, University of Gothenburg; Clinical Neurochemistry Laboratory (H.Z., K.B.), Sahlgrenska University Hospital, Mölndal, Sweden; Department of Molecular Neuroscience (H.Z.), UCL Institute of Neurology; Trauma and Neuroscience Centre (R.A., A.M.), Blizard Institute, Barts and the London School of Medicine and Dentistry, Queen Mary University of London; and MRC Centre for Neuromuscular Diseases (M.L., M.M.R., A.M.R.), UCL Institute of Neurology and National Hospital for Neurology and Neurosurgery, London, UK.

## Abstract

**Objective:**

To perform a cross-sectional study to determine whether plasma neurofilament light chain (NfL) concentration is elevated in patients with Charcot-Marie-Tooth disease (CMT) and if it correlates with disease severity.

**Methods:**

Blood samples were collected from 75 patients with CMT and 67 age-matched healthy controls over a 1-year period. Disease severity was measured using the Rasch modified CMT Examination and neuropathy scores. Plasma NfL concentration was measured using an in-house-developed Simoa assay.

**Results:**

Plasma NfL concentration was significantly higher in patients with CMT (median 26.0 pg/mL) compared to healthy controls (median 14.6 pg/mL, *p* < 0.0001) and correlated with disease severity as measured using the Rasch modified CMT examination (*r* = 0.43, *p* < 0.0001) and neuropathy (*r* = 0.37, *p* = 0.044) scores. Concentrations were also significantly higher when subdividing patients by genetic subtype (*CMT1A*, *SPTLC1*, and *GJB1*) or into demyelinating or axonal forms compared to healthy controls.

**Conclusion:**

There are currently no validated blood biomarkers for peripheral neuropathy. The significantly raised plasma NfL concentration in patients with CMT and its correlation with disease severity suggest that plasma NfL holds promise as a biomarker of disease activity, not only for inherited neuropathies but for peripheral neuropathy in general.

Peripheral neuropathy affects an estimated 10 million people in the European Union (7 million in the United States) and represents a substantial public health burden.^1,2^ Nerve conduction studies are the gold standard for confirming a large-fiber peripheral neuropathy; however, in severe cases, such studies may be unable to monitor disease progression due to a floor effect. There is therefore a need for a biomarker that reflects peripheral nerve damage and can be used to assess response to treatment.

Neurofilaments are the major cytoskeletal proteins of neurons in both the CNS and peripheral nervous system and form a lattice comprising neurofilament light (NfL), medium, and heavy (NfH) chains.^3^ Damage to nerves leads to the release of these proteins into the CSF or plasma, as demonstrated by the 20-year-old observation that NfL concentration is increased in the CSF of patients with amyotrophic lateral sclerosis (ALS).^4^ Increased NfL concentrations have been reported in CSF, and more recently in plasma, in neurodegenerative diseases of the CNS; for example, frontotemporal dementia (FTD), multiple sclerosis (MS), and Alzheimer disease (AD).^5–8^

In contrast to diseases of the CNS, biomarkers of peripheral nerve damage are less well-developed. In this study, we sought to determine the biomarker potential of plasma NfL in peripheral neuropathy by measuring the plasma NfL concentration using ultrasensitive Simoa immunoassay technology,^9^ in patients with both demyelinating and axonal forms of inherited neuropathy (Charcot-Marie-Tooth disease [CMT]), a group of diseases in which patients experience slowly progressive axonal degeneration at a constant rate.^10^ A blood biomarker of disease activity is likely to be of value in future treatment trials in CMT, a disease that often shows only minimal progression during the usual 1- to 2-year period of a clinical trial and in which current clinical outcome measures are insensitive in detecting disease progression during such a time period.^11^

## Methods

### Participants

Blood samples were collected prospectively, with informed consent, from all patients with CMT with a genetically confirmed diagnosis attending the inherited neuropathy clinic at the National Hospital for Neurology and Neurosurgery, London, between January 2011 and May 2013. Patients with other neurologic diseases were excluded as determined by review of the clinical notes and a questionnaire. The disease severity, as measured using the Rasch modified CMT examination score, version 2^12^ (the weighted CMTES), was recorded at the same time as plasma was collected. The weighted CMTES is a validated outcome measure for assessing the severity of CMT. It is a composite score that includes the patient's symptoms and examination findings.^11^ All patients underwent nerve conduction studies to confirm the presence of a neuropathy; however, a weighted CMT neuropathy score (CMTNS) (which required neurophysiology at the same time as the clinical assessment) was only included if a nerve conduction study had been performed within 18 months of the blood sample.

Blood samples were obtained from healthy relatives of patients attending the inherited neuropathy clinic and staff at the UCL Institute of Neurology and from the relatives of patients with ALS recruited as part of a separate study. Healthy controls were excluded if they had coexistent neurologic disease as determined by a symptom and medical history–based questionnaire.

### Blood sampling and sample collection and storage

All participants were evaluated in outpatient clinics and blood samples were taken and processed within 1 hour. Whenever possible, a repeat blood sample was taken after 1 year. Blood was collected into EDTA-containing tubes and centrifuged at 20°C at 3,500 rpm for 10 minutes. Plasma was then aliquoted and stored at −80°C.

### Standard protocol approvals, registrations, and patient consents

This study was approved by The National Hospital for Neurology and Neurosurgery Research Ethics Committee/Central London REC 3 09/H0716/61 and the East London and the City Research Ethics Committee 1 (09/H0703/27). Written informed consent was obtained from all participants in the study.

### Simoa plasma NfL measurements

Plasma sample NfL concentration was determined using the in-house Simoa NfL assay, which has been described in detail previously.^7^ Briefly, paramagnetic carboxylated beads (Quanterix, Boston, MA) were coated with a mouse anti-NfL antibody (UD1; UmanDiagnostics, Umeå, Sweden) and incubated for 35 minutes with sample and a biotinylated mouse anti-NfL antibody (UD2; UmanDiagnostics) in a Simoa HD-1 instrument (Quanterix). The bead-conjugated immunocomplex was thoroughly washed before incubation with streptavidin-conjugated β-galactosidase (Quanterix). After additional washes, resorufin β-d-galactopyranoside (Quanterix) was added and the immunocomplex was applied to a multiwell array designed to enable imaging of every single bead. The average number of enzymes per bead (AEB) of samples was interpolated onto the calibrator curve constructed by AEB measurements on bovine NfL (UmanDiagnostics) serially diluted in assay diluent. Samples were analyzed blind and in duplicate using one batch of reagents. The average repeatability coefficient of variation of a sample with the mean concentration 14.3 pg/mL was 7.5% and the interassay coefficient of variation was 12%, and for a sample with a mean concentration of 129.5 pg/mL this was 5.3% and 10.2%, respectively. The limit of detection, determined as the mean blank signal +3 SD for the Simoa NfL assay, was 0.3 pg/mL, and the lower limit of quantification (LLOQ), determined as the mean blank signal +10 SD, was 2.7 pg/mL when compensated for a 4-fold sample dilution. All samples analyzed were above the LLOQ.

### Statistical analysis

Statistical analysis was performed using SPSS version 23.00 (IBM; Armonk, NY) and GraphPad Prism 5.0 (GraphPad Inc., La Jolla, CA). A normal probability plot of the residuals of plasma NfL concentration revealed a skewed distribution of the data points. Plasma NfL concentration was therefore compared using a 2-sided Mann-Whitney *U* test, and correlations were assessed using Spearman and Pearson correlation coefficients. Fisher *r* to *z* transformation was used to compare correlation coefficients. Multiple linear regression was performed using SPSS to model the relationship between plasma NfL (dependent variable) and age and weighted CMTES score (independent variables).

## Results

### Patient demographics

A total of 75 patients with CMT (including 21 with hereditary sensory neuropathy due to mutations in *SPTLC1* and *SPTLC2*) and 67 healthy controls were enrolled into the study. There was no significant difference in the mean age of the 2 groups (table 1; *p* = 0.73). There were significantly more women in the control (69%) than the CMT cohort (52%, *p* = 0.043); however, this is unlikely to have influenced the comparison between the 2 cohorts as there was no significant difference in plasma NfL concentration between male and female controls (14.0 [10.9–20.5] vs 14.9 [10.9–25.3] pg/mL, *p* = 0.829). The CMT cohort comprised 10 different subtypes of CMT. The commonest subgroup was CMT1A (n = 31), followed by SPTLC1 (n = 20) and CMTX1 (n = 11). Hereditary sensory neuropathy due to mutations in *SPTLC1* was disproportionately represented in this study as samples were collected from patients recruited into a natural history study of HSN1 that was running concurrently. Sixty-four percent of patients with CMT had CMT1 (demyelinating neuropathy) vs 36% with CMT2 (axonal neuropathy), which is reflective of the relative prevalence of these disease subtypes in the general population.^13^ All patients included in the study had a weighted CMTES calculated; however, only 30 (40%) had a neurophysiologic examination within 18 months of sample collection and for whom a weighted CMTNS was calculated.

**Table 1:**
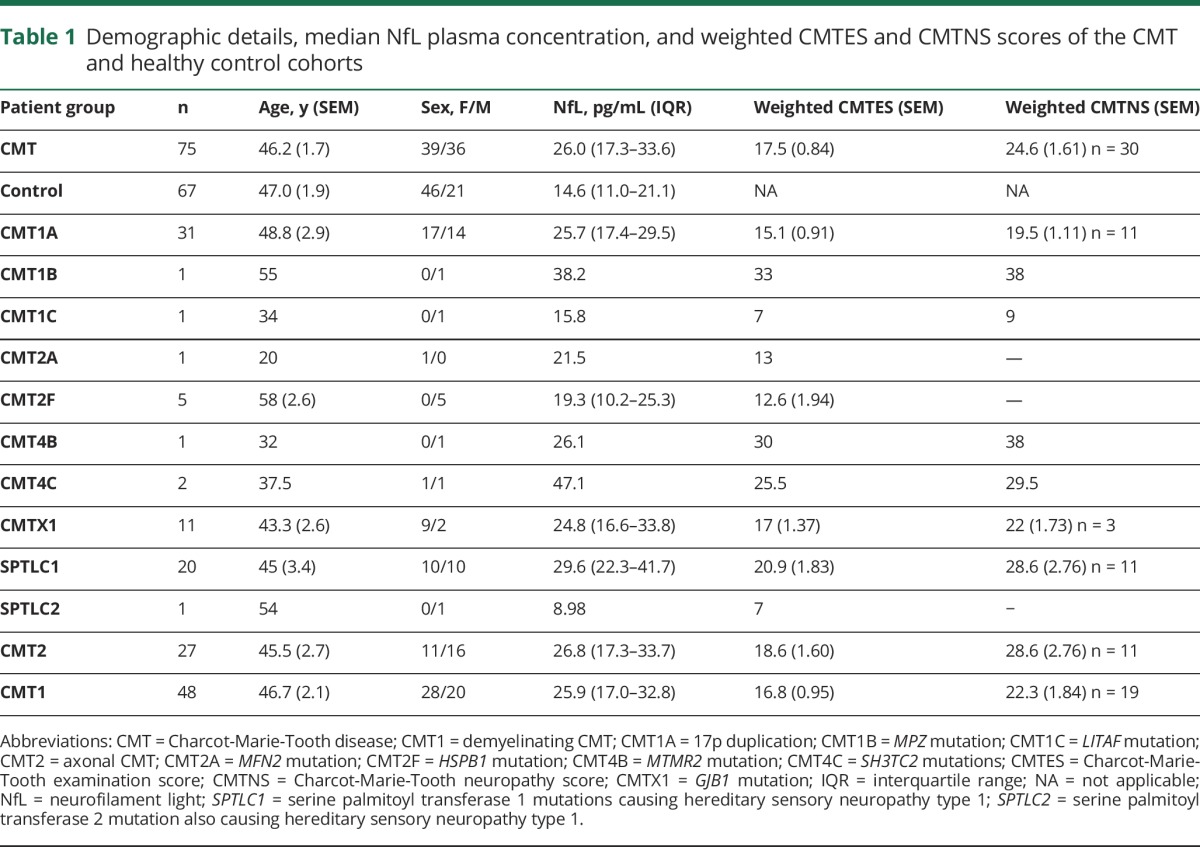
Demographic details, median NfL plasma concentration, and weighted CMTES and CMTNS scores of the CMT and healthy control cohorts

### Plasma NfL chain concentration is increased in patients with inherited peripheral neuropathy

**Figure 1:**
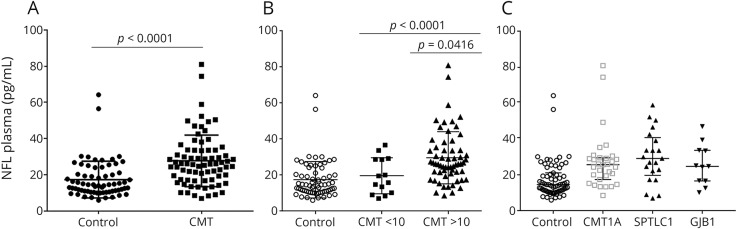
Plasma neurofilament light (NfL) concentration in Charcot-Marie-Tooth disease (CMT) (A) Significantly increased plasma NfL concentration in patients with CMT (n = 75) compared to healthy controls (n = 67). (B) Significantly increased plasma NfL concentration in patients with more severe disease (weighted CMT examination score [CMTES] >10) vs milder CMT (weighted CMTES <10) and healthy controls. (C) Plasma NfL concentration for the 3 largest subgroups of CMT, CMT1A (n = 31), hereditary sensory neuropathy due to mutations in *SPTLC1* (n = 20), and CMTX1 (n = 11).

Plasma NfL concentration was significantly higher in patients with CMT compared to age-matched healthy controls (*p* < 0.0001; table 1 and figure 1A). Furthermore, patients with more severe disease classified as a weighted CMTES >10 had higher median NfL concentration compared to patients with a CMTES <10 (*p* = 0.0416; figure 1B). The significant difference in plasma NfL concentration compared to controls was maintained for individual comparisons of the 3 common genetic subtypes included in this study (*CMT1A*: *p* < 0.0001; *SPTLC1*: *p* < 0.001; *GJB1*: *p* = 0.011, figure 1C). Furthermore, plasma NfL concentration was also increased in individual patients with CMT1B, CMT2A, CMT4B2, and CMT4C compared to controls (table 1).

### Plasma NfL concentration increases with advancing age

**Figure 2:**
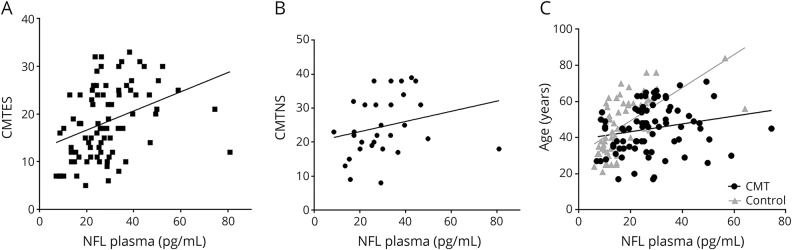
Plasma neurofilament light (NfL) concentration and Charcot-Marie-Tooth disease (CMT) severity (A) Significant correlation between disease severity as measured by the weighted CMT examination score (CMTES) and plasma NfL concentration. (B) Significant correlation between plasma NfL concentration and disease severity as measured by the weighted CMT neuropathy score (CMTNS). (C) Significant correlation between plasma NfL concentration and age, which is more pronounced for healthy controls.

Plasma NfL concentration correlated with age and this was more pronounced in controls than in patients with CMT (control: *r* = 0.70, *p* < 0.0001; CMT: *r* = 0.28, *p* = 0.012; Fisher *r* to *z* transformation (*p* < 0.0001); figure 2C). This is unlikely to have confounded our analysis as the CMT and control group were age-matched (table 1).

### Plasma NfL concentration correlates with disease severity in patients with inherited neuropathy

The weighted CMTES and CMTNS are disease severity scales for peripheral neuropathy that have been modified to create a linear scale. The CMTES includes a symptom and examination score; the CMTNS in addition includes neurophysiologic data. A comparison of the weighted CMTES and plasma NfL concentration revealed a significant correlation between NfL concentration and disease severity (*r* = 0.43, *p* < 0.0001, figure 2A). This correlation was even more positive if the single outlier seen in figure 1 was removed (*r* = 0.46, *p* < 0.0001). The outlier is a patient with CMT1A and a weighted CMTES score of 12 but a very high plasma NfL concentration of 81.0 pg/mL. A retrospective clinical review of this patient (6 years after sample collection) did not reveal an alternative cause for the high concentration. The correlation between plasma NfL concentration and disease severity remained significant when correlated against the weighted CMTNS (*r* = 0.37, *p* = 0.044), although this correlation could only be performed for the 40% of patients who had a weighted CMTNS calculated (figure 2B). If the prominent outlier was excluded from the analysis, the correlation coefficient was 0.46 (*p* = 0.012). When plasma NfL concentration was predicted using a multiple linear regression model with age and the weighted CMTES as independent variables, it was found that disease severity as measured using the weighted CMTES was a significant predictor (β = 0.34, *p* < 0.003), whereas age was not (β = 0.15, *p* = 0.17). The overall model fit was *R*^2^ = 0.17. There was no significant correlation between plasma NfL concentration and the ulnar compound muscle action potential (CMAP) (Pearson *r* = −0.227, *p* = 0.228).

### Plasma NfL concentration is stable over 1 year in patients with CMT

**Figure 3:**
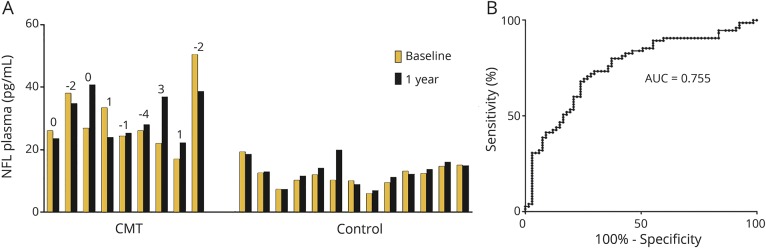
Plasma neurofilament light (NfL) concentration as a diagnostic biomarker of Charcot-Marie-Tooth disease (CMT) (A) The individual NfL concentration at baseline and after 1 year for all patients with CMT (n = 9) and healthy controls (n = 13). Above the bars is the individual change in weighted CMT over 1 year. There is no significant difference in plasma NfL concentration in either patients with CMT or healthy controls after 1 year. (B) Receiver operator curve of NfL concentration for detecting patients with peripheral neuropathy. AUC = area under the curve.

On a selected number of patients with CMT (n = 9) and controls (n = 13), plasma samples were collected at baseline and after 1 year. There was no significant difference in plasma NfL concentration in patients with CMT (mean difference −1.07 pg/mL, 95% confidence interval −8.2 to 6.0 pg/mL; paired *t* test *p* = 0.74) or controls (mean difference +1.19 pg/mL, −0.45 to 2.8, *p* = 0.14) when comparing baseline with 1-year follow-up samples (figure 3A). The NfL concentration coefficient of variability over 1 year was 16.4% in patients with CMT and 9.2% in controls, which is similar to the interassay variation (5%–12%) of Simoa NfL measurements. There was no significant change in the weighted CMTES at 1 year follow-up (mean difference −0.44, *p* = 0.5).

### Plasma NfL concentration discriminates patients with CMT from healthy controls

To assess the ability of plasma NfL to discriminate patients with inherited peripheral neuropathy from controls, we plotted a receiver operator characteristic curve (figure 3B), which revealed an area under the curve of 0.755. A comparison of the sensitivity and specificity for a range of cutoff values suggested that a concentration of 20 pg/mL identifies patients with inherited peripheral neuropathy with a sensitivity of 71% and specificity of 75% (table 2).

**Table 2:**
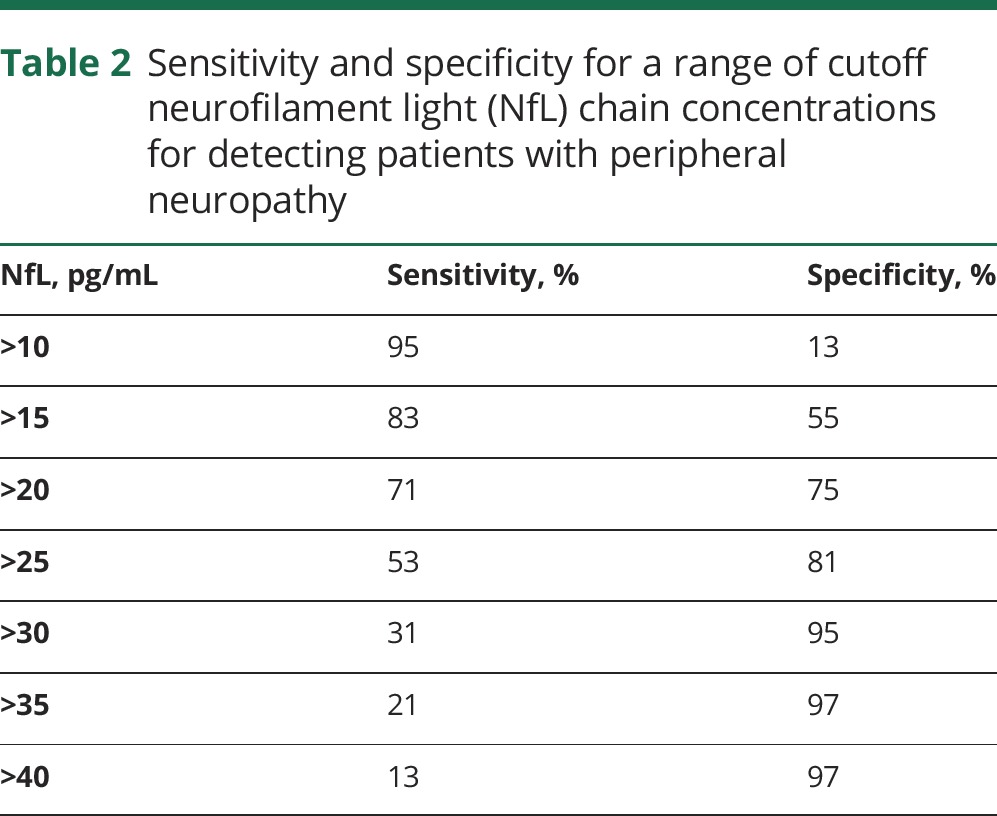
Sensitivity and specificity for a range of cutoff neurofilament light (NfL) chain concentrations for detecting patients with peripheral neuropathy

## Discussion

In this study, we have demonstrated that plasma NfL concentration is increased in patients with inherited peripheral neuropathy and that it correlates with disease severity. It is important to emphasize that raised plasma NfL concentration was not limited to CMT1A, but was significantly elevated in the 2 other forms of CMT with sufficient numbers of participants for analysis (CMT1X and HSN1). This suggests a role for plasma NfL in monitoring disease activity may be extended to multiple forms of CMT. Further studies are therefore warranted to discover if these results can be replicated for other common forms of CMT such as CMT1B and CMT4C. The observation of raised plasma NfL in individual patients with these subtypes in this study is encouraging.

Increased plasma NfL concentration is not specific to CMT as a similar increase has been reported in several other neurologic disorders such as ALS, MS, FTD, and AD.^5–7,14^ Therefore, plasma NfL concentration will not by itself be useful for diagnosis; however, it may provide a dynamic measure of axonal damage and serve as a biomarker of disease activity for future clinical trials and monitoring response to treatment, especially in the many conditions like CMT where neuropathy is the only neurologic manifestation with no CNS involvement and therefore any change in NfL levels would reflect neuropathy changes alone. As neurofilaments are almost exclusively expressed in neurons, there are likely to be few non-neurologic confounding factors; however, neurofilaments are expressed in T lymphocytes and theoretically the concentration may be falsely elevated in T-cell proliferative disorders.^11^

We have previously measured plasma NfH concentration in the same cohort of patients and controls and were unable to detect a difference or a correlation with disease severity.^15^ This is unsurprising and reflects similar paradoxical results in cohorts of patients with ALS^16^ and may reflect the formation of NfH aggregates resulting in falsely low levels.^17^ In this study, we were also unable to detect a correlation between the ulnar CMAP and plasma NfL concentration. One possible explanation may be that our sample population included many individuals with mild CMT (CMTES <10) in whom the ulnar CMAP may be normal.

Our observation that plasma NfL concentration correlates with disease severity in CMT suggests that plasma NfL may also show promise as a biomarker of disease activity. The CMT neuropathy and examination scores are clinical outcome measures based on a patient's symptoms, clinical examination, and neurophysiology and are currently the gold standard for measuring disease severity for patients with CMT.^18^ There are, however, several limitations to the score, including a quasi-linear scale and a ceiling effect for severe patients.^18^ A blood biomarker of axonal damage and thereby disease activity is likely to be of considerable value in future treatment trials in CMT and other peripheral neuropathies that show only minimal progression during the usual 1- to 2-year period of a clinical trial. To date there have been 4 large randomized clinical trials in patients with the commonest form of CMT, CMT1A, comparing ascorbic acid to placebo.^19–22^ All 4 trials failed to show a therapeutic benefit of ascorbic acid, but perhaps equally as important, the first version of the CMTNS, which was used as the primary outcome measure in 3 of the 4 trials, was unable to detect disease progression over a 1-year period.^18^ This is important, as treatments for neurodegenerative diseases such as CMT are at best likely to stop progression. Encouragingly, quantitative MRI has been shown to be sensitive to detecting an increase in the fat fraction of muscle in patients with CMT1A over a 1-year period, suggesting its suitability as a primary outcome measure for future treatment trials.^23^ The sensitivity of muscle MRI, however, is dependent on the choice of muscle, whereby severely affected and unaffected muscles are susceptible to both ceiling and floor effects. A similar floor effect is also seen with nerve conduction studies, where in a proportion of patients with moderate to severe peripheral neuropathy, the nerves are so severely damaged that no electrical response can be recorded. In this regard, a blood biomarker has the advantage of reflecting damage to all nerves and is likely to be more sensitive to multifocal peripheral nerve diseases such as vasculitis.

Although neurofilaments are axonal cytoskeletal proteins, plasma concentration was similarly raised in both axonal and demyelinating forms of CMT. This is perhaps unsurprising in view of the importance of Schwann cell axonal interaction in neuronal maintenance and previous observations that the degree of disability in demyelinating CMT1A is due to the degree of axonal loss rather than the degree of conduction velocity slowing (a surrogate marker of demyelination).^24^ This finding also suggests that plasma NfL concentration may show promise as a biomarker of disease activity in other demyelinating peripheral neuropathies such as Guillain-Barré syndrome and chronic inflammatory demyelinating polyneuropathy.

We collected follow-up samples from 9 individuals with CMT at 1 year, revealing an intrasubject variability of plasma NfL concentration of 16.4%. As one would expect for a genetic disease such as CMT in which there is constant but slow progression, implying a constant level of peripheral axonal degeneration, we could not detect a change in plasma NfL concentration over 1 year. The plasma concentration of NfL is likely to be dependent on the rate of production (i.e., axonal degeneration), the volume of distribution, and the half-life. A successful treatment for peripheral neuropathy should lead to a reduction in axonal degeneration and a decrease in plasma NfL concentration. Further studies will be required to assess the sensitivity of plasma NfL for detecting individual temporal changes in axonal degeneration over time.

In this study, we have shown that plasma NfL concentration is sensitive to detecting peripheral axonal damage in CMT, that it correlates with disease severity, and that there is little intrasubject variability. We have not demonstrated the responsiveness of plasma NfL concentration to change in the rate of axonal degeneration, a vital step in assessing the suitability of plasma NfL concentration in monitoring response to therapy in peripheral neuropathy and as a biomarker for treatment trials in CMT. Further studies addressing this point are currently in progress.

## Glossary

AD: Alzheimer disease
AEB: average number of enzymes per bead
ALS: amyotrophic lateral sclerosis
CMAP: compound muscle action potential
CMT: Charcot-Marie-Tooth disease
CMTES: Charcot-Marie-Tooth examination score
CMTNS: Charcot-Marie-Tooth neuropathy score
FTD: frontotemporal dementia
LLOQ: lower limit of quantification
MS: multiple sclerosis
NfH: neurofilament heavy
NfL: neurofilament light
